# Substance use among young people in sub-Saharan Africa: a systematic review and meta-analysis

**DOI:** 10.3389/fpsyt.2024.1328318

**Published:** 2024-09-11

**Authors:** Jemal Ebrahim, Jon Adams, Daniel Demant

**Affiliations:** ^1^ School of Public Health, Faculty of Health, University of Technology Sydney, Sydney, NSW, Australia; ^2^ Department of Psychiatry, Faculty of Health Sciences, Madda Walabu University, Shashemene, Ethiopia; ^3^ School of Public Health and Social Work, Faculty of Health, Queensland University of Technology, Brisbane, QLD, Australia

**Keywords:** substance use, substance use problems, substance use disorders, alcohol use, drug use, khat use, sub-Saharan Africa, young people

## Abstract

**Background:**

The use of substances such as alcohol, tobacco, khat, or drugs among young people is becoming a public health concern globally, with particularly high prevalence rates in low and middle-income settings, where socio-cultural and economic factors contribute to distinct challenges in addressing this problem. This review aimed to summarize the current literature on the prevalence of substance use among young people in sub-Saharan Africa (SSA) and identify gaps in the current body of literature.

**Methods:**

Seven databases and Google were searched for studies reporting on substance use prevalence among young people (aged 10-24 years) in SSA, published between January 2010 and May 2024. Observational studies were included, assessed for methodological quality, and checked for the presence of heterogeneity and publication bias using standard methods. A random effect model was used to estimate the pooled proportions for substance use among young people.

**Results:**

The literature search identified 1,889 hits from the databases and Google. Among these 60 eligible studies involving 83,859 respondents were included in the review. The overall lifetime, 12-month, and current prevalence of any substance use among young people in SSA was found to be 21.0% (95% CI= 18.0, 24.0), 18% (95% CI=10,27), and 15% (95% CI=12,18), respectively. Among young people from SSA, alcohol use problem was the most prevalent (40%), followed by khat use (25%), stimulant use (20%), and cigarette smoking (16%). Other substances used by a smaller proportion of young people included cannabis, cocaine, inhalants, sedatives, shisha, hallucinogens, steroids, and mastics. The prevalence of substance use problems was higher among males compared to females, highest in the southern African region followed by Western and Eastern regions, and in community-based studies compared to institutional-based studies.

**Conclusions:**

In SSA, over a fourth of young people use at least one substance in their lifetime, with higher rates among males than females and in community-based compared to institution-based studies. These results emphasize the need for interventions targeting the wider young population and those in specific subgroups identified as being at higher risk of substance use. This approach allows for the provision of tailored support and resources to those who need it most while also promoting positive health outcomes for the entire population of young people in the region.

**Systematic Review Registration:**

https://www.crd.york.ac.uk/prospero/display_record.php?ID=CRD42022366774, identifier CRD42022366774.

## Introduction

Young people, defined as individuals aged 10-24 years (1), constitute a significant portion of the global population (24%), estimated at approximately 1.9 billion (2). This demographic is primarily concentrated in low- and middle-income countries (LMICs), where 90% of young people reside (2). Sub-Saharan Africa (SSA) is home to one of the largest and fastest-growing populations of young people, with individuals in this age group making up over 60% of the region’s total population (3). This demographic presents both a potential for economic growth and a challenge in terms of public health and social issues, including substance use (4).

Substance use among young people is a critical public health concern globally, significantly contributing to the burden of disease (5). The Global Burden of Disease (GBD) study identifies substance use disorders (SUDs) as major contributors to morbidity and mortality among young people (6). In SSA, the situation is particularly alarming (7–9). Recent data indicates a rising trend in substance use among adolescents in this region, with substantial implications for their health and well-being (7, 10).

Several risk factors contribute to substance use among young people. These include socio-economic factors such as poverty, unemployment, and lack of education, as well as social and environmental influences like peer pressure, family dynamics, and the availability of substances (11, 12). Additionally, psychological factors such as stress, trauma, and mental health play a significant role (13, 14). Understanding these risk indicators is crucial for developing effective prevention and intervention strategies.

The consequences of substance use among young people are profound and multifaceted. Substance use is associated with a range of risky behaviors, including unprotected sex, violence, and criminal activities (8, 15), directly and indirectly leading to an increase in the likelihood of poor health outcomes, such as sexually transmitted infections (STIs), injuries, and mental health disorders (15–17). Furthermore, substance use can lead to chronic conditions, contributing to long-term morbidity and premature mortality (18, 19). The impact extends beyond individual health, affecting families, communities, and broader societal structures (20).

The present review aimed to address the need for a comprehensive understanding of substance use among young people in SSA. While previous reviews have provided valuable insights in this area, some of them focused on specific countries (12, 21, 22), while others focused on adolescents (10-19 years of age) (10), or on specific substances of use (23, 24). This systematic review and meta-analysis sought to fill the gap by offering a region-wide perspective on the prevalence and risk factors of substance use among young people (covering both adolescents and youth) in SSA. By addressing these gaps, the study will contribute to the understanding of substance use in SSA, informing policy and programmatic responses to mitigate this pressing public health issue among young people.

## Materials and methods

### Protocol and registration

This review was prepared and reported in accordance with the Preferred Reporting Items for Systematic Reviews and Meta-Analyses (PRISMA) 2015 guideline (25). The protocol was registered on the International Prospective Register of Systematic Reviews under CRD42022366774.

### Search strategy

Studies published between January 1, 2010, and May 31, 2024, were searched through a comprehensive search in the following databases: African Journals Online (AJOL), PubMed, ProQuest, PsycINFO, Web of Science, Scopus, and African Index Medicus (AIM) library. The chosen databases are widely used for indexing publications in health and substance use, have search functions appropriate for conducting systematic reviews, are generally perceived as trustworthy within academia, and index a large number of journals that include work published in our geographic area of interest (SSA). The body of knowledge in the fields of health and behavioral sciences changes quickly with the acquisition of new knowledge, discoveries, theories, processes, or best practices, and there is a need to share the most recent evidence with practitioners in those fields. Accordingly, our systematic review aimed to provide a synthesis of recent evidence published in the last 14 years in the area of substance use for practitioners, policymakers, clinical practice guideline developers, program designers, and those designing and justifying primary research.

Additional searches were made using the Google search engines, including using the reference lists of identified original research. Comprehensive search terms were designed using Medical Education Subject Heading (MeSH) and keywords, were primarily developed for the PubMed search protocol and applied for other databases as well. Details of the search terms and strategies used are included in the **Supplementary Material** (**Supplementary Material S1**).

### Inclusion and exclusion criteria

This review considered all observational study designs, including case-control, cohort, and cross-sectional studies, that empirically investigated the prevalence and patterns of substance use among young individuals aged 10-24 years. Young people were defined as those from 10 to 24 years of age, encompassing both adolescents and youths (1, 26). Studies published in English between January 1, 2010, and May 31, 2024, and involving human subjects were eligible for inclusion. The review excluded anonymous reports, letters, editorials, brief communications, comments, and reviews.

### Study selection

The articles retrieved were initially loaded into the EndNote program, where authors identified and removed the duplicates. Subsequently, the remaining articles were exported to Covidence for title, abstract, and full-text screening. Each screening stage adhered to the predetermined eligibility criteria. JE conducted the screening, with ongoing discussions among the authors to ensure consistency and consensus at each step. Any minor disagreements that arose in the screening and review process were resolved by discussion.

### Data extraction

A standardized data extraction format was adapted from the Joanna Briggs Institute (JBI) data extraction format (27), and implemented in a Microsoft Excel sheet version 365. The data extracted included the following information: author/s, publication year, study location (country), study design, sample size, gender distribution, participant age, types of substances used, prevalence of substance use, tools used to measure substance use, associated factors, and common reasons for use substance (**Table 1**). JE extracted the required study parameters or information from the included studies, with all authors closely collaborating at each step.

**Table 1:** Summary characteristics of studies included in the systematic review and meta-analysis of the prevalence of any substance use among young people in sub-Saharan Africa.

S.N	Author, year	Country	Design	Setting	Age range	Sample size	Gender (M/F)	Type substance studied	Combined prevalence		
									Lifetime	12-month	30-days
1.	Ogunkunle et al., 2020 (28)	Nigeria	Cross-sectional	Community-based	10-18	501	259/242	Cigarettes, Alcohol, Cannabis, Stimulant, Sedatives, Codeine cough syrup, Tramadol, Cocaine, Hallucinogens, solvents/inhalants	0.09		0.06
2.	Gobopamang et al., 2016 (29)	Botswana	Cross-sectional	Institution based	10-19	3567	1,565/2002	Tobacco, Alcohol, Marijuana, Glue, Mandrax, Cocaine, Ecstasy, Sextasy, others	0.10	0.52	0.07
3.	Teni et al., 2015 (30)	Ethiopia	Cross-sectional	Institution based	16-30	400	239/161	Khat	0.42		0.32
4.	Johnson et al., 2017 (31)	Nigeria	Cross-sectional	Institution based	18-25	324	170/154	Alcohol, codeine, tramadol, cigarette, Marijuana, Steroid	0.56		
5.	Hamdulay & Mash R., 2011 (32)	South Africa	Cross-sectional	Institution based	≥16 and <16	438	209/229	Alcohol, tobacco smoking, cannabis, crystal methamphetamine, ecstasy, mandrax, solvents, and cocaine.	0.19	0.12	0.10
6.	Kassa et al., 2014 (33)	Ethiopia	Cross-sectional	Institution based	15-30	586	479/107	Alcohol, khat, cigarettes, marijuana, and other illicit PASs such as Ecstasy, lysergic diethylamide (LSD), cocaine, crack, heroin, solvents or inhalants, and un-prescribed psychoactive medications	0.09	0.18	
7.	Siziya et al., 2013 (34)	Zambia	Cross-sectional	Institution based	13-16+	2257	994/1039	Cannabis	0.40		
8.	Dida et al., 2014 (35)	Ethiopia	Cross-sectional	Institution based	15-29	603	373/230	Khat, Alcohol, Cigarette, Shisha			0.13
9.	Abdeta et al., 2017 (36)	Ethiopia	Cross-sectional	Institution based	20-25	619	464/155	khat	0.26		0.10
10.	Osayomi et al., 2011 (37)	Nigeria	Cross-sectional	Institution based	14-18	243	243/-	Non-amphetamine stimulants, alcoholic beverages, Inhalants, Sedatives or sleeping pills, Heroin, morphine, pain medication, Tobacco products, Marijuana, Hallucinogens, Cocaine or crack, Amphetamine-type stimulants	0.08		0.07
11.	Tsegaye A. et al., 2021 (38)	Ethiopia	Cross-sectional	Institution based	17-25, ≧̸25	409	206/203	khat	0.24		0.22
12.	Bright A. et al., 2016 (39)	Ghana	Cross-sectional	Institution based	14-19 years	240	119/121	Alcohol, Marijuana, Tobacco	0.20		
13.	Adere et al., 2017 (40)	Ethiopia	Cross-sectional	Institution based	18-25	655	444/211	Alcohol drinking, khat chewing, and cigarette smoking.	0.18	0.17	0.15
14.	Admasu et al., 2018 (41)	Ethiopia	Cross-sectional	Institution based	18-25	403	274/129	Khat	0.12		0.10
15.	Alebachew et al., 2019 (42)	Ethiopia	Cross-sectional	Institution based	18-26	251	171/80	Khat. Cigarette smoking, Alcohol drinking, and other substances (hashish, cocaine, cannabis, etc.)	0.26		0.18
16.	Astatkie et al., 2015 (43)	Ethiopia	Cross-sectional	Institution based		1252	927/325	Khat	0.44	0.16	0.11
17.	Ayenew et al., 2020 (44)	Ethiopia	Cross-sectional	Community-based	12–18 years	312	281/31	Khat, Cigarette, Mastics, Alcohol, Benzene	0.26		0.28
18.	Birhanu et al., 2014 (45)	Ethiopia	Cross-sectional	Institution based	14-19	651	358/293	Alcohol, cigarettes, and khat	0.39		0.21
19.	Chekole, 2020 (46)	Ethiopia	Cross-sectional	Institution based	15-30 years	803	537/266	Alcohol	0.31		0.24
20.	Chivandire, 2016 (47)	Zimbabwe	Cross-sectional	Institution based	13-19	311	180/131	Cannabis	0.16		0.07
21.	Deressa, 2011 (48)	Ethiopia	Cross-sectional	Institution based	18-39 years	622	426/196	Alcohol, cigarette, khat chewing	0.18	0.14	0.05
22.	Desai et al., 2019 (49)	South Africa	Cross-sectional	Community-based	13-20	4185	2506/1716	Cigarette smoking			0.50
23.	Dires et al., 2016 (50)	Ethiopia	Cross-sectional	Institution based	13-23	296	168/128	Khat	0.16		0.14
24.	Durowade et al., 2021 (51)	Nigeria	Cross-sectional	Institution based	20-29	416	228/188	Alcohol, cigarettes, marijuana, cannabis, opioids, methamphetamine,			0.18
25.	Francis et al., 2015 (52)	Tanzania	Cross-sectional	Mixed	15-24 yrs	1954	1264/690	Alcohol	0.51	0.27	0.13
26.	Gebrehanna et al., 2014 (53)	Ethiopia	Cross-sectional	Institution based		3001	2328/673	Khat	0.02	0.13	0.08
27.	Gebremariam et al., 2018 (54)	Ethiopia	Cross-sectional	Institution based	15-25 years	617	363/254	Alcohol, khat, cigarette, cannabis, cocaine, and heroin	0.11		0.09
28.	Gebresilassie et al., 2020 (55)	Ethiopia	Cross-sectional	Institution based	18-29 years	1207	889/318	Alcohol, khat, cigarette, cannabis, cocaine	0.25		0.13
29.	Gebreslassie et al., 2013 (56)	Ethiopia	Cross-sectional	Institution based	15-30 years	756	444/312	Khat, Alcohol, Cigarette	0.24	0.24	0.23
30.	Getachew et al., 2019 (57)	Ethiopia	Cross-sectional	Community-based	13–19 years	3932	2117/1815	Alcohol and tobacco	0.19		0.08
31.	Hirpa et al., 2021 (58)	Ethiopia	Cross-sectional	Institution based	13-22 years	3319	1515/1804	Shisha smoking	0.02		0.01
32.	Itanyi et al., 2020 (59)	Nigeria	Cross-sectional	Institution based	10-19 years	4332	1889/244	Tobacco products			0.13
33.	Kanyoni et al., 2015 (60)	Rwanda	Cross-sectional	Community-based	14-35 years	2479	1388/1091	Alcohol, tobacco smoking, cannabis, glue, and drugs such as diazepam.	0.13	0.10	0.09
34.	Kassa et al., 2017 (61)	Ethiopia	Cross-sectional	Community-based	10-24 years	1577	860/717	Khat	0.15		0.06
35.	Kassa et al., 2016 (62)	Ethiopia	Cross-sectional	Institution based	15-30 year	586	479/107	Alcohol and Khat	0.36	0.31	0.23
36.	Kuteesa et al., 2020 (63)	Uganda	Cross-sectional	Community-based	15-24 year	1281	675/606	Alcohol and other illicit drugs	0.35		
37.	Mayanja et al., 2020 (64)	Uganda	Cross-sectional	Community-based	15-24 year	1440	-/1440	Alcohol			0.75
38.	Musyoka et al., 2020 (65)	Kenya	Cross-sectional	Community-based		406	206//200	Alcohol, Tobacco, Cannabis, Others (opioids, cocaine, amphetamine, hallucinogens, sedatives, and inhalants.)	0.12		
39.	Mutiso et al., 2022 (66)	Kenya	Cross-sectional	Institution based	15-43 years	9673	5173/4500	Opioids	0.03		0.09
40.	Ogunsola et al., 2016 (67)	Nigeria	Cross-sectional	Institution based	10-19 years	600	345/255	Heroin, cocaine, cannabis, tobacco, sniffing substances, alcoholic beverages	0.16		0.03
41.	Olashore et al., 2018 (68)	Botswana	Cross-sectional	Institution based	18-24 years	401	199/202	Alcohol, tobacco, cannabis, Inhalants, LSD, cocaine, heroin, petrol and glue, ATS, methylphenidate, street drugs, crystal methamphetamine, khat, and benzodiazepines.			0.11
42.	Onya et al., 2012 (69)	South Africa	Cross-sectional	Institution based	11-25 years	1600	744/856	Alcohol	0.15		
43.	Oshodi et al., 2010 (70)	Nigeria	Cross-sectional	Institution based	11-20 years	402	175/227	Tobacco, alcohol, cannabis, opiates, cocaine, psychostimulants, hallucinogens, organic solvents, and hypnosedatives.	0.27	0.07	0.17
44.	Owusu-Sarpong et al., 2019 (71)	Ghana	Cross-sectional	Institution based	12 -19 years	700	336/364	Cigarette Smoking	0.18		
45.	Reda et al., 2012 (72)	Ethiopia	Cross-sectional	Institution based	15-25 years	1707	856/851	Khat	0.27		
46.	Riva et al., 2018 (73)	Botswana	Cross-sectional	Institution based	14-17 years	1933	904/1029	Alcohol and other drugs	0.25		
47.	Roble et al., 2021 (74)	Ethiopia	Cross-sectional	Community-based		341	243/98	Cigarette smoking	0.23		0.21
48.	Seid et al., 2021 (75)	Ethiopia	Cross-sectional	Institution based	15-24 years	374	180/194	Alcohol, Khat, cigarette, cannabis, Shisha	0.14		0.06
49.	Shegute et al., 2021 (76)	Ethiopia	Cross-sectional	Institution based	15-30 years	782	468/314	Alcohol, Khat, cigarettes, and other illicit drugs	0.56		0.37
50.	Sinshaw et al., 2014 (77)	Ethiopia	Cross-sectional	Institution based	18-23 years	302	183/119	Khat	0.10		0.07
51.	Soepnel et al., 2022 (78)	South Africa	Cross-sectional	Community-based	18-28 years	1508	-/1508	Tobacco use	0.51		
52.	Soremekun et al., 2020 (79)	Nigeria	Cross-sectional	Institution based	10–15 years	1048	533/515	Tobacco, Alcohol, Cannabis, Cocaine, Inhaled things, Tranquilisers, Sedatives, Heroin, Opioids (pharmaceutical)	0.07	0.02	0.03
53.	Augustus et al., 2024 (80)	Sierra Leone	Cross-sectional	School-based	10–19 years	1730	845/885	Alcohol, cannabis	0.04		0.11
54.	Einarsdóttir, 2024 (81)	Guinea-Bissau	Cross-sectional	School-based	14–19 years	2039	1015/1024	Shisha, tobacco, alcohol, cannabis	0.18		0.15
55.	Jaguga et al., 2023 (82)	Kenya	Mixed method	Youth-clinic based	16–19 years	100	59/41	Tobacco, alcohol, cannabis, cocaine, khat, sedatives, hallucinogen	0.12		
56.	Kinyanjui et al., 2023 (83)	Kenya	Cross-sectional	University based	median 21	400	203/197	Alcohol, cigarettes, cannabis, sedatives, khat, tranquilizers, inhalants, heroin, opium, cocaine	0.07		
57.	Kyei-Gyamfi, 2024 (84)	Ghana	Mixed method	Community-based	10–17 years	4144	2099/2045	Alcohol, cigarettes, cannabis, tramadol, codeine, shisha, heroin, cocaine	0.13		
58.	Kyei-Gyamfi, 2023 (85)	Ghana	Mixed method	Community-based	8–17 years	5024	2566/2458	Alcohol	0.07		
59.	Mavura, 2022 (86)	Tanzania	Cross-sectional	School-based	10–19 years	3224	1515/1709	Cigarettes, alcohol, khat, marijuana, amphetamine	0.05		0.03
60.	Olashore, 2022 (87)	Botswana	Cross-sectional	School-based	12–19 years	742	326/407	Alcohol, tobacco, inhalants, cannabis, cocaine, ATS, LSD, sedatives, solvents, heroin		0.12	

### Quality appraisal

The quality assessment was primarily done by JE and DD, supplemented via discussions among the authors. The JBI Meta-Analysis of Statistics Assessment and Review Instrument (JBI-MAStARI) quality assessment tool (27) was used to assess quality. JBI-MAStARI tools are among the most commonly used scales for assessing quality, and risk of bias for cross-sectional, cohort, case-control, qualitative, and observational studies (88) (**Supplementary Material S2**). The appraisal aims to assess the methodological robustness of a study and its effectiveness in addressing the potential bias in the design, conduct, and analysis. All papers chosen for the review were subject to an appraisal based on the scoring guide. Scores were awarded for each criterion as follows: 0 = not fulfilling the criteria; 1 = fulfilling the criteria. The scores of each criterion added up and the result ranged from 0- 9. Those studies with a score above the mean for methodological quality appraisal were included in the review. The scoring relied on JBI guidance notes (88) as well as the judgment and expertise of our review team. Studies with a score below the mean were considered unfit and excluded from the review. Features considered to report as low quality include unmentioned study sample size determination, insufficient sample size, and absence of statistical evaluation on the reliability and validity of relevant assessment tools among others (**Supplementary Material S3**).

### Data analysis and synthesis

The results of this review were organized to include descriptive characteristics of the study, a narrative synthesis of findings organized by subgroups, and a meta-analysis result.

The meta-analysis was conducted using Stata Version 18 statistical software to estimate the effect sizes using a standard error (SE) and 95% confidence interval (CI). To estimate the overall prevalence of substance use, the lifetime, one-year, and current prevalence data from each study were combined, along with their corresponding SE. Lifetime prevalence was defined as the use of a substance at least once in an individual’s lifetime, 12-month prevalence was the use of a substance at any time during the past year, and current prevalence was the use of substances within the past 30 days before data collection. Accounting for the potential overlap between the different substance uses, the combined (overall) prevalence from each study were computed first. Then, the overall prevalence in the three reporting patterns (lifetime, current, and 12 months) was analyzed separately using the combined prevalence and their corresponding SE.

A random effect model, which accounts for potential heterogeneity (89), was used to estimate the pooled effects in the meta-analysis. A forest plot was utilized to illustrate the combined estimate with the 95% CI. Heterogeneity among the studies was assessed through visual inspection of the forest plots and quantified using the I² statistic (90, 91). Heterogeneity levels were classified as low (I² = 25%), moderate (I² = 50%), and high (I² = 75%) based on I² statistics (91). Publication bias was assessed using Egger’s test, with a p-value of less than 0.05 indicating possible publication bias (91).

Furthermore, to minimize random variations between each primary study, sub-group analysis was undertaken by the region and country where a study collected empirical data (east, south, and west Africa); sex of study participants (male, female); year of publication; as well as the study setting type (community-based versus institution-based). These variables were selected due to their inclusion in the majority of reviewed studies.

## Results

### Search results

**Figure 1:**
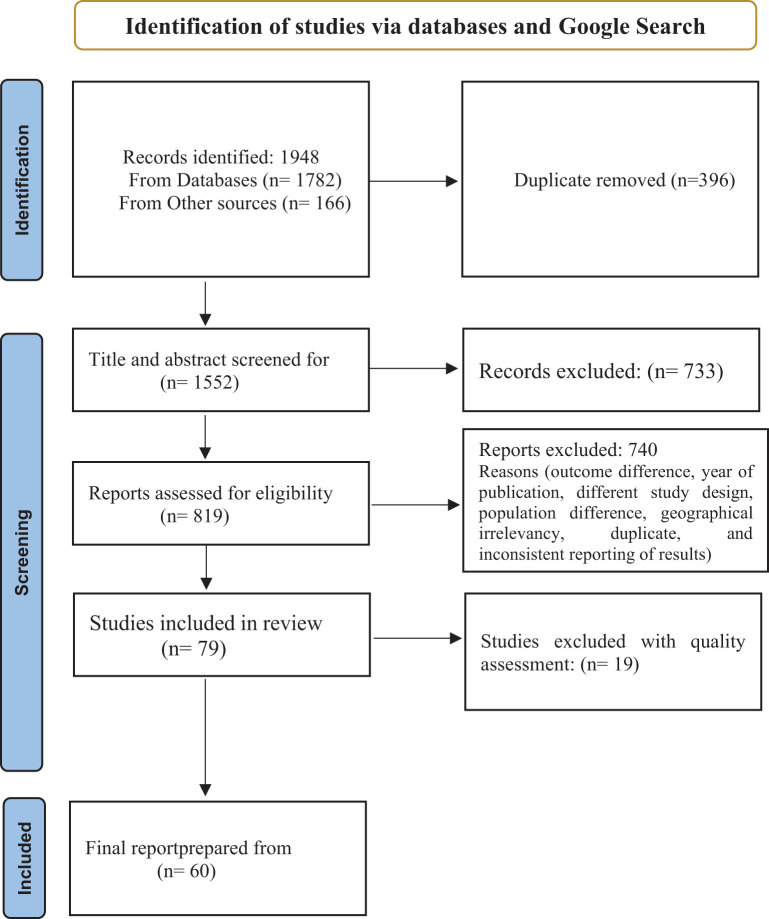
Flow diagram of the studies included in the systematic review and meta-analysis of substance use among young people in Sub-Saharan Africa.

The search yielded 1,948 hits from the databases with an additional 166 articles identified through Google searches. After excluding duplicates and studies that did not meet the review inclusion criteria, 79 studies were eligible for review. Out of these, 19 were judged to be of poor methodological quality and excluded. The types of features deemed to present low methodological quality were: no mention of the study sample size determination, insufficient sample size, and a lack of statistical assessment regarding the reliability and validity of relevant measurement tools among others. The remaining 60 were considered for full review (see **Figure 1**).

### General characteristics of the reviewed studies

The total sample size across the included studies was 83,859 respondents, with 54.0% male, and 46.0% female. Sample sizes in individual studies ranged from 100 (82) to 9,742 (66) participants.

The review considered studies focused on the prevalence of substance use (lifetime, 12-month, and current) among young individuals, with almost all being cross-sectional empirical studies.

**Figure 2:**
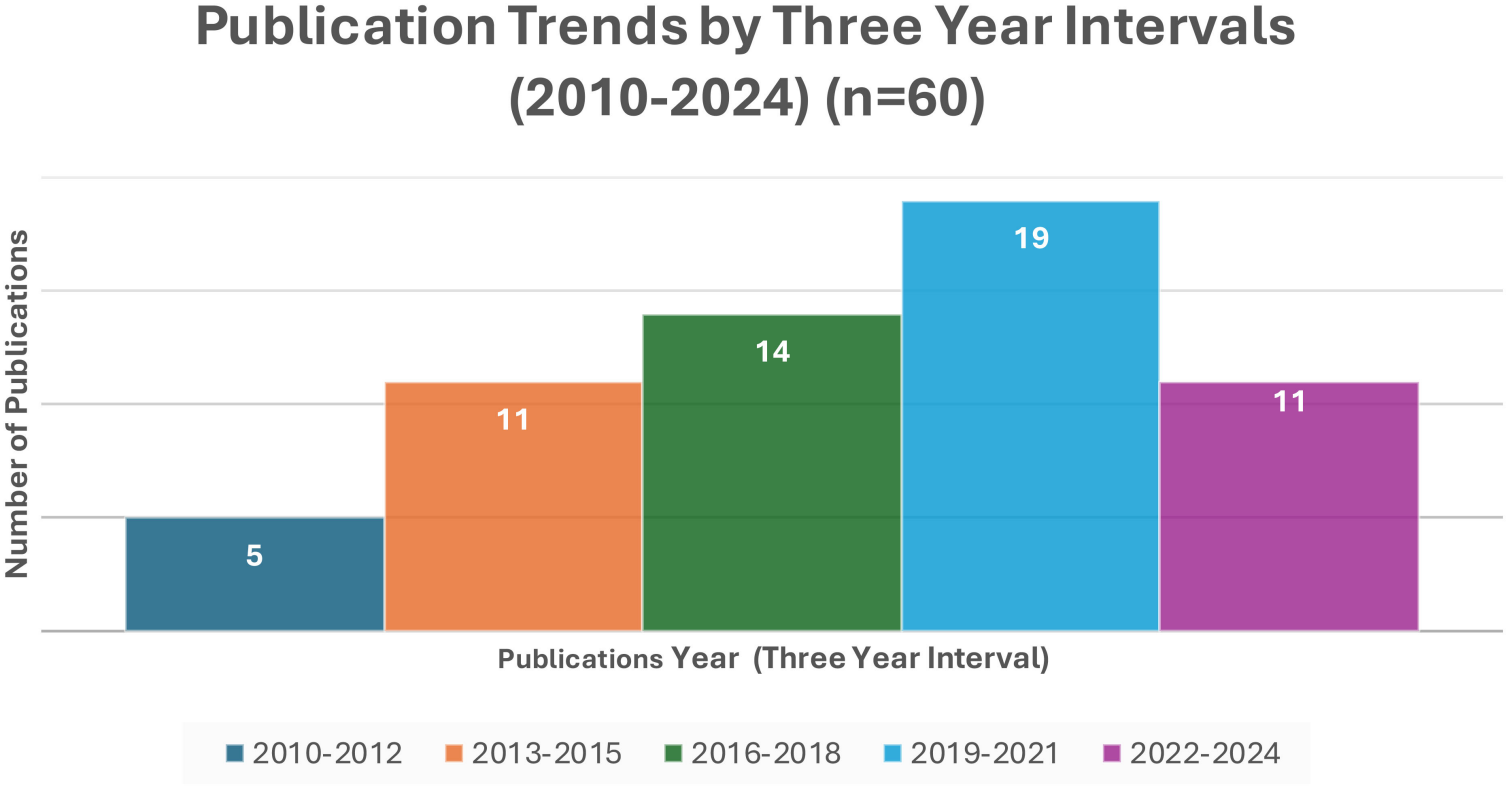
Trends in the publication of substance use studies in three years interval from 2010-2024.

Concerning publication trends, **Figure 2** shows a relative increase in publication trends on substance use over the last five years in SSA.

As shown in **Table 1**, among the reviewed studies, alcohol was the most frequently reported (n=41), followed by cigarette smoking (n=36), khat use (n=28), and cannabis use (n=26), among other substances.

Most studies were conducted in educational institutions, with forty-six studies in schools, universities, and student clinics, and thirteen studies in community settings. Additionally, one study had a mixed population from schools, colleges, local industries, and casual laborers (see **Table 1**).

To measure substance use, 24 (40.0%) studies employed standardized tools such as the Alcohol, Smoking and Substance Involvement Screening Test (92) (ASSIST), CAGE (93), Alcohol Use Disorders Identification Test (AUDIT) (94), Cannabis Abuse Screening Test (CAST) (95), and the Global Student Drug Use Survey questionnaire (96), while 36 (60.0%) studies used questionnaires adapted from previous literature.

### Prevalence of substance use

In general, the prevalence of substance use varied widely among studies, ranging from 2% (95% CI=1.0, 2.0) (58) in a study from Ethiopia to 56.0% (95% CI=51.0, 62.0) (31) in a study from Nigeria.

#### Lifetime prevalence

**Figure 3:**
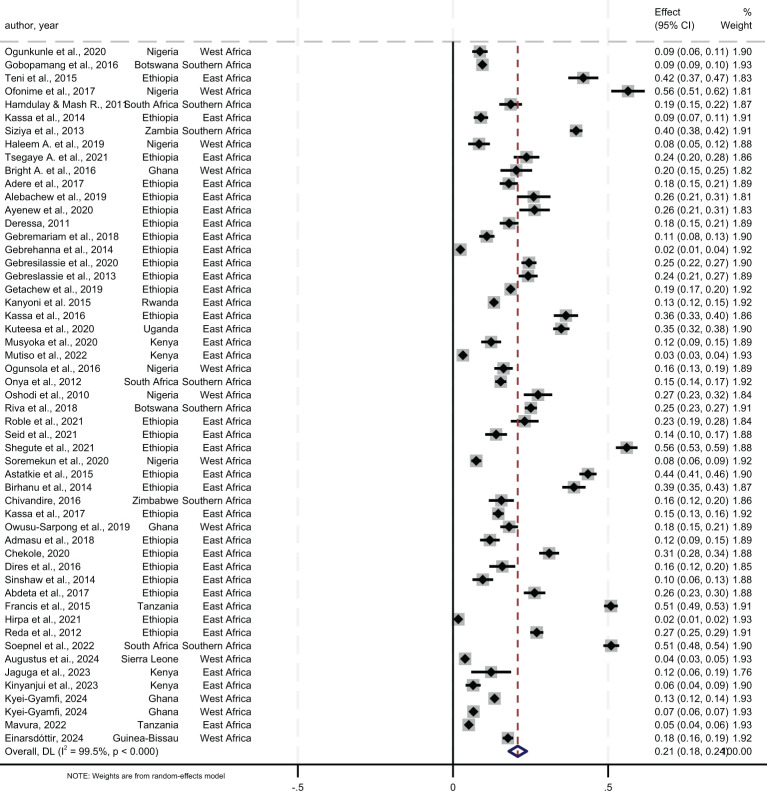
Pooled lifetime prevalence of any substance use among young people in sub-Saharan Africa, 2024.

**Figure 4:**
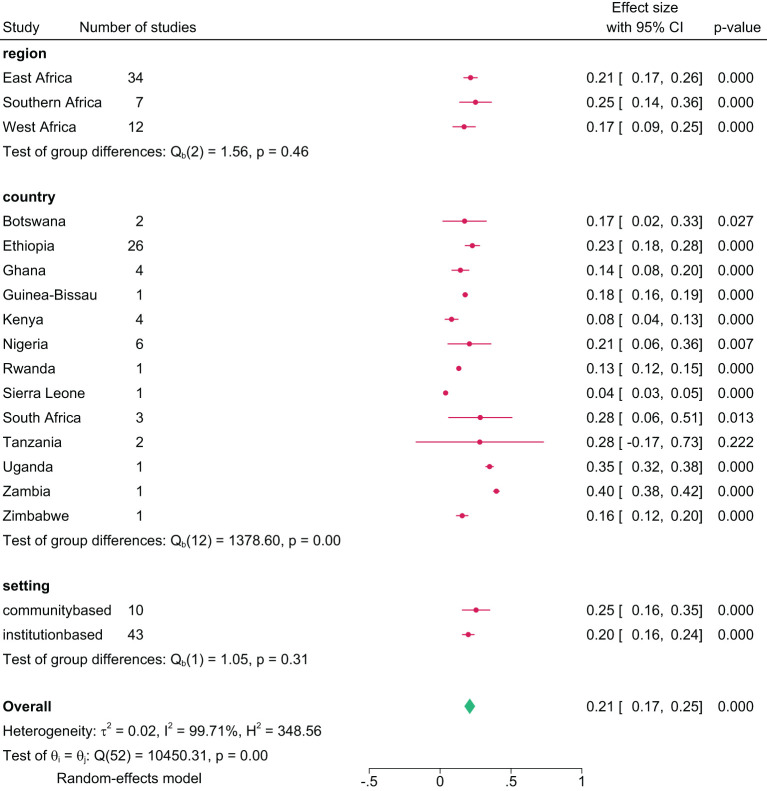
Pooled lifetime prevalence of any substance use among young people in sub-Saharan Africa by subgroup, 2024.

The lifetime prevalence of any substance use, pooled from 53 studies, was 21.0% (95% CI= 18.0, 24.0) (**Figure 3**). Regionally, Southern Africa had the highest lifetime prevalence at 25.0% (95% CI=13.0, 37.0), followed by East Africa at 22.0% (95% CI=18.0, 26.0), and West Africa at 17.0% (95% CI=13.0, 21.0). Country-specific data showed Zambia with the highest lifetime prevalence at 40.0% (95% CI=38.0, 42.0) and Sierra Leone the lowest at 4.0% (95% CI=3.0, 5.0). Analysis by study setting where participants were recruited showed a pooled lifetime prevalence of 20.0% in both community and institutional settings (**Figure 4**).

#### Current prevalence

**Figure 5:**
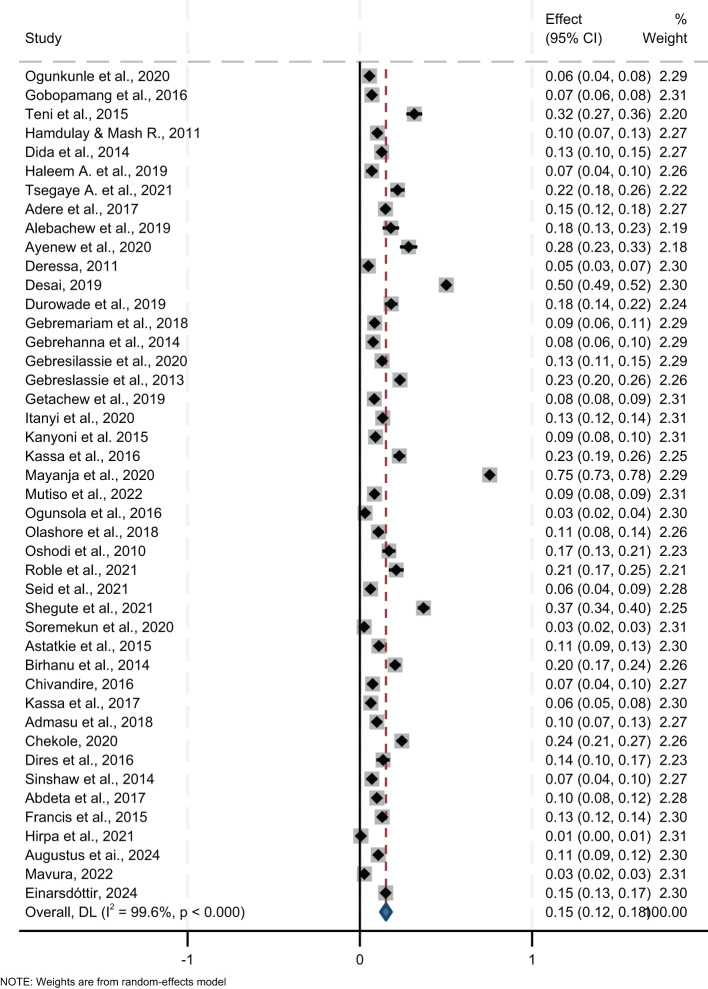
Pooled current prevalence of any substance use among young people in sub-Saharan Africa, 2024.

**Figure 6:**
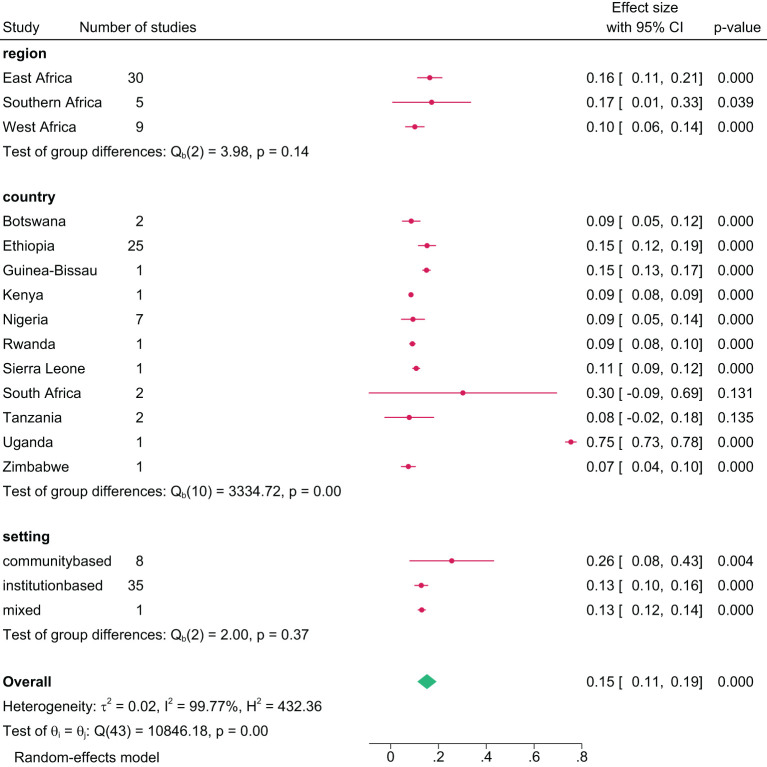
Pooled current prevalence of any substance use among young people in sub-Saharan Africa by subgroup, 2024.

The pooled current prevalence of any substance use was 15.0% (95% CI=12.0, 18.0), with the lowest at 2.5% (95% CI=1.6, 3.4) (79) and the highest at 75.0% (95% CI=73.0, 78.0) (64) from individual studies (**Figure 5**). In a further analysis by region, East Africa had the highest current prevalence at 17.4% (95% CI=13.0, 22.0), followed by Southern Africa, 17.1% (95% CI=3.0, 37.0), and West Africa at 10.0% (95% CI=6.0, 14.0). Among countries, the highest current prevalence was recorded in Uganda at 75.0% (95% CI=73.0, 78.0) and the lowest in Zimbabwe at 7.0% (95% CI=5.0, 10.0). Additional analysis by study setting showed a pooled current prevalence of 15.0% (95% CI=11.0, 19.0), with 26% from community-based studies and 13.0% from institution-based studies (**Figure 6**).

#### 12-month prevalence

**Figure 7:**
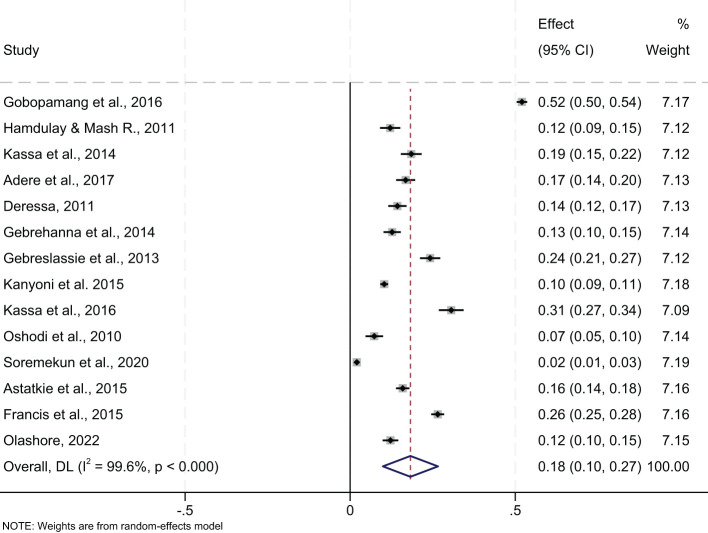
Pooled 12-months prevalence of any substance use among young people in sub-Saharan Africa, 2024.

**Figure 8:**
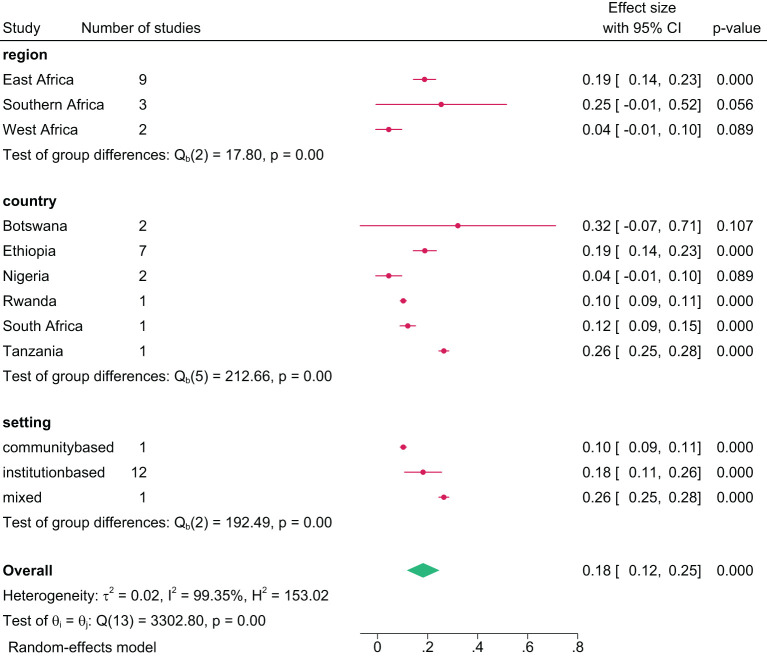
Pooled 12-months prevalence of any substance use among young people in sub-Saharan Africa by subgroup, 2024.

The 12-month prevalence of any substance, pooled from 14 studies, was 18.0% (95% CI=10.0, 27.0) (**Figure 7**). Botswana had the highest 12-month prevalence at 32.0% (95% CI=7.0, 71.0), and Nigeria lowest at 4.0% (95% CI=1.0, 10.0). Further group analysis by region showed the lowest 12-month prevalence in West Africa at 4.0% (95% CI=1.0, 10.0), and the highest in Southern Africa at 25% (95% CI=4.0, 55.0) (**Figure 8**). The review showed a pooled 12-month prevalence of 18.0% (95% CI=11.0, 26.0) in institution-based studies and 10.0% (95% CI=9.0, 11.0) in community-based studies (**Figure 8**).

### Prevalence of specific substances

#### Alcohol consumption

The lifetime prevalence of alcohol use, pooled from 39 studies, was 36.2% (95% CI=29.4, 43.0). The prevalence was higher in Uganda at 51.4% (95% CI=48.3, 53.7), followed closely by Rwanda, at 50.6% (95% CI= 49.0, 53.0), while it was comparatively lower in Botswana at 29.0% (95% CI=3.5, 54.5). Alcohol prevalence in individual studies ranged from 2.0% (95% CI=2.0, 2.0) (58) to 69.0% (95% CI=67.0, 71.0) (78). Furthermore, gender-disaggregated data from 19 studies showed a higher lifetime prevalence among males, 45.9% (95% CI=32.5, 59.4) compared to females, 25.6% (95% CI=18.6, 32.7).

The pooled current prevalence of alcohol use, based on 35 studies, was 23.6% (95% CI=19.1, 28.2). Among studies with gender-disaggregated data, 10 reported a prevalence of 48.7% (95% CI=27.8, 32.7) among males and 14.3% (95% CI=9.9, 18.7) among females.

The pooled 12-month prevalence of alcohol use, based on 10 studies, was 30.0% (95% CI=17.0, 44.0). Similarly, pooled data from 10 studies on 12-month prevalence showed 58.9% (95% CI=40.8, 76.9) among males and 25.0% (95% CI=16.4, 33.7) among females. The highest 12-month alcohol use prevalence was recorded in South Africa at 41.0% (95% CI=36.4, 45,6), and the lowest in Nigeria at 3.0% (95% CI=0.3, 0.9).

#### Cigarette use

The pooled lifetime prevalence of cigarette use, from 36 studies, was 15.2% (95% CI=12.5, 17.9). Country-specific analysis showed highest lifetime cigarette use in South Africa at 30.4% (95% CI=7.6, 53.2) and lowest in Tanzania at 7.6% (95% CI=6.7, 8.5). Prevalence reported by individual studies ranged from 1.0% (95% CI=0.1, 2.0) (41) to 49.7% (95% CI=45.0, 54.4) (78). Gender-disaggregated data from 14 studies showed a higher lifetime prevalence in males at 36.6% (95% CI=15.4, 57.7) compared to females at 10.7% (95% CI=7.2, 14.3).

The pooled current prevalence of cigarette use, based on 31 studies, was 11.8% (95% CI=8.8, 14.8). Among these, 9 studies provided gender-disaggregated data, showing a current prevalence of 27.7% (95% CI=11.1, 44.3) in males and 8.1% (95% CI=3.1, 13.2) in females. By country, South Africa had a higher current prevalence at 42.7% (95% CI=28.0, 57.4), while Tanzania had a lower prevalence at 4.0% (95% CI=3.3, 4.7).

For 12-month prevalence, the pooled estimate from 12 studies was 12.5% (95% CI=7.8, 17.2). Regionally, data from 7 studies showed a higher 12-month prevalence in South Africa at 36% (95% CI=31.0, 41.0) and lower in West Africa at 2.0% (95% CI=0.1, 4.0). Additionally, 3 studies (32, 40, 56) reported 12-month prevalence rates of 50.0% (95% CI=0.1, 100.0) among males and 22.5% (95% CI=2.0, 43.0) among females.

#### Khat use

The pooled lifetime prevalence of khat use, from 28 studies, was 23.0% (95% CI=16.9, 29.1). Country-specific analysis showed higher lifetime khat consumption in Ethiopia at 25.8% (95% CI=20.3, 31.3) and lower in Tanzania at 2.0% (95% CI=1.2, 2.8). The lifetime prevalence reported by individual studies ranged from 2.0% (95% CI=1.2, 2.8) (86) to 63.0% (95% CI=58.3, 67.7) (44). The current and 12-month prevalence of khat was 17.3% (95% CI=13.4, 21.1) and 16.0% (95% CI:16.0, 21.0), respectively. Generally, the lifetime, 12-month, and current prevalence of khat use were higher among males than females: 51.0% (95% CI: 31.0, 70.0), 48.0% (95% CI: 12.0, 84.0), and 51.0% (95% CI: 31.0, 71.0) for males, compared to 13% (95% CI: 9.0, 17.0), 9% (95% CI: 5.0, 13.0), and 14% (95% CI: 10.0, 18.0) for females, respectively.

#### Cannabis use

The pooled lifetime prevalence of cannabis use was 11.0% (95% CI=9.0, 13.0), with the highest recorded in Zambia at 37.0% (95% CI=35.0, 39.0) and the lowest in Tanzania at 1.0% (95% CI=0.7, 1.3). The lifetime prevalence reports from individual studies ranged from 0.5% (95% CI=0.1, 2.0) (41) to 61.0% (95% CI=58.0, 64.0) (43). Among nine studies with gender-disaggregated data, the pooled lifetime prevalence of cannabis use was 26.0% (95% CI=3.0, 48.0) among males and 18.0% (95% CI=10.0, 26.0) among females. The current pooled prevalence of cannabis use, based on 17 studies, was found to be 3.7% (95% CI=2.7, 4.7), while the 12-month prevalence, from 5 studies, was 6.0% (95% CI=3.0, 9.0).

#### Cocaine use

Twelve studies in the review reported a lifetime cocaine use prevalence of 3.0% (95% CI=2.0, 3.0) among young people. The highest prevalence was recorded in Botswana at 6.0% (95% CI=5.3, 6.7) (29), and the lowest was in South Africa at 1.0% (95% CI=0.1, 1.9) (32). The lifetime prevalence of cocaine use reported in individual studies range from 1.0% (95% CI=0.1, 2.0) (32) to 6.0% (95% CI=5.0, 7.0) (29). For the current and 12-month period, the prevalence of cocaine use was 0.8% (95% CI=0.4, 1.1) and 1.3% (95% CI=0.4, 2.3) respectively.

#### Shisha use

Seven studies (33, 54, 58, 72, 74, 81, 84) reported the lifetime prevalence of shisha use, and four studies (35, 36, 58, 81) reported the current prevalence. The pooled lifetime prevalence was 8.4% (95% CI=5.4, 11.8), and the pooled current prevalence was 6.2% (95% CI:0.3, 12.2).

#### Hallucinogens

This group includes studies reporting on hallucinogens and LSD (Lysergic Acid Diethylamide). The lifetime prevalence of hallucinogen pooled from 6 studies, was 3.0% (95% CI=1.0, 5.0), ranging from 1.0% in Kenya (82) to 7.0% in Ethiopia (44). Only one study from Ethiopia (44) reported a lifetime prevalence of 0.5% among males and 3.0% among females. The current prevalence of hallucinogens, based on 3 studies (32, 44, 97), was found to be 2.2% (95% CI=0.7, 3.6).

#### Sedatives

Fifteen studies reported on sedative use prevalence (tranquilizers, sedatives, and Mandrax). The pooled lifetime prevalence of sedative use was 8.9% (95% CI=7.0, 10.8), with individual study reports ranging from 0.1% (95% CI=0.1, 0.2) in Rwanda (60) to 73.8% (95% CI=69.3, 78.3) (97) in Nigeria. Country-specific analysis showed the highest lifetime prevalence in Nigeria at 19.0% (95% CI=9.8, 28.1), and the lowest in Rwanda at 0.1% (95% CI=0.1, 0.2). The current prevalence of sedative use, pooled from 10 studies, was 6.2% (95% CI=4.7, 7.8), while the 12-month prevalence, based on 4 studies, was 6.0% (95% CI=3.0, 10.0).

#### Opioid group

Of the studies included in the meta-analysis, sixteen reported on an opioid group of drug use among young people. The pooled lifetime prevalence of opioids was 15.9% (95% CI=13.2, 18.5). The current prevalence of opioid use, pooled from 10 studies, was 7.0% (95% CI=5.0, 9.0), while the 12-month prevalence, from 3 studies, was 4.0% (95% CI=1.0, 7.0).

#### Inhalants group

A total of ten studies reported on inhalant (e.g., benzene, glue, petrol, and solvents) use among young people. The pooled lifetime prevalence of inhalants, from 12 studies, was 6.0% (95% CI=4.0, 8.0). The lowest reported prevalence from an individual study was in Botswana at 0.1% (95% CI=0.01, 0.2) (29), while the highest was 7.40% (95% CI=4.1, 10.7) (97) in Nigeria.

The current prevalence of inhalant use, pooled from 9 studies, was 3.0% (95% CI=2.0, 4.0), while the 12-month prevalence, from 4 studies was 2.0% (95% CI=0.1, 4.0).

#### Other substances (steroids and mastics)

Two studies reported on this group of substance use, one focusing on mastics in Ethiopia (44) and the other is on steroids in Nigeria (31). The current prevalence of mastics was 42.0% (95% CI=36.5, 47.5), and the lifetime prevalence was 46.0% (95% CI=40.5, 51.5) (44). The lifetime prevalence of steroids was reported at 12.2% (95% CI=7.8, 14.6) (31).

### Factors associated with substance use

Out of the 60 studies reviewed, only 48 reported on factors associated with substance use among young people. For simplicity, these factors were grouped into three major categories: individual, family, and community and environmental factors.

#### Individual factors

Among the studies providing gender distribution data, 18 consistently show a higher prevalence of substance use among male young individuals (29, 30, 33, 35, 36, 38, 40, 42, 48, 50, 53–56, 61, 65, 66, 84). In contrast, while three studies report a higher prevalence of substance use among females (31, 34, 51), two studies indicate lower rates among females (57, 87).

Ten reviewed studies indicate that substance use risk increases with age among young people (31, 44, 50, 55, 57, 63, 66, 72, 84, 85). However, a Zambian study on cannabis use found younger individuals, below 15 years, showing higher rates than older ones (34).

It was indicated in the reviewed studies that the risk of substance use among young people increases with advancing years of study (29, 38, 40, 43, 46, 55, 76, 79). Additionally, factors such as poor academic performance (45), non-attendance of school (44, 71), attending private schools (54, 67), attending rural schools (59), and in-school compared to out-of-school young people (28) are associated with an increased risk of substance use. However, one study presents a different perspective, indicating a lower risk of substance use among high-academic performers (31).

Furthermore, factors such as living alone during school-age (33, 62), living off-campus in rented accommodation (43, 53, 65), low perceived risk of substance use (45), poor social skills (45), engagement in sexual activity (47, 50, 61, 63), being bullied (47, 87), and the perception that substance use improves academic achievement (53) were linked to various forms of substance use among young people.

Additionally, urban residency (55, 76), frequent watching of football games or soccer matches (57), having internet access at home (57), experiencing intimate partner violence (63), involvement in trade and other business activities (71, 84, 85), exposure to substance advertisements (87), and the presence of health conditions such as anxiety symptoms (68, 76), human immunodeficiency virus (63), suicidal ideation (50, 73), and low expectations for the future (73) were also associated with substance use among young people.

#### Family factors

Peer and family influence significantly impact substance use among young people. As reported by 17 studies in the review, substance use by close friends (31, 33, 35, 38, 41, 43–47, 53, 54, 56, 57, 67, 68, 71) and other family members (21, 31, 33, 36, 43, 47, 54, 56, 68, 71, 75) were associated with substance use among young people.

Young people with negative family dynamics, such as unsatisfactory family relationships (31), parental divorce (39, 76), low family monitoring (73), loss of one or more parents (76), and those who felt poorly understood by their parents (87) had higher odds of substance use. Conversely, a strong parent-child relationship (75), parental disapproval of substance use (67), and close family supervision (34) were associated with a lower tendency to use substances among young people. Additionally, young people whose mothers had secondary or higher education (51, 60, 67), or were civil servants (51) had a higher prevalence of substance use.

#### Community and environmental factors

Community norms supportive of substance use (75), easy access to substances (75, 79), and lenient school regulations (75, 79) were associated with substance use among young people.

Frequent attendance at religious places (30, 36, 45, 54), regardless of the type of religion, was associated with a lower risk of substance use among young people, while never attending was associated with an increased risk (69).

While two studies indicate low socioeconomic status increases the risk (68, 78), eight other reviewed studies showed higher odds of substance use among individuals from higher socioeconomic and income backgrounds compared to their counterparts (21, 30, 40, 42, 54, 58, 59, 79).

### Reasons for substance use among young people

A total of twenty-one studies reported reasons for substance use among young people. Common reasons included alertness (28, 33, 50, 61, 75, 76), boosting confidence (31, 41, 47, 84, 85), exam preparation (28, 36, 38, 41, 43, 50, 70), peer influence (33, 36, 38, 41, 44, 50, 61, 74–76) (51), increasing energy (33, 43), and stress relief (33, 36, 38, 41, 43, 47, 50, 51, 61, 70, 74–76, 84).

Other reported reasons were pleasure/fun (33, 36, 38, 41, 43, 46, 47, 50, 61, 70, 74–76, 84), stomachache relief (33), family influence (33, 36, 76), easy access (36, 38, 44, 75, 76) and socialization (36, 41–44, 46, 50, 76).

Additional reasons included enhanced concentration on religious activities (36, 42, 43, 70), appetite suppression (36, 50), time-killing (41, 43), improving academic performance (41), habit (43, 61), lack of recreational areas (42), affordability (44), curiosity (33, 44, 51, 70, 74) fitting in with friends (47, 76), media influence (51), self-medication (70, 85), having money to buy (75), academic dissatisfaction (76), improving social status (85), and stimulating appetite for meals (85).

## Discussion

This review provides a comprehensive overview of substance use prevalence and risk factors among young people in SSA, pooled from 60 different individual studies published between 2010 to 2024. While previous reviews often focused on specific countries (12, 21, 22), adolescents (10-19 years of age) (10), or specific substances (23, 24) have provided valuable insights, this review offers a broader regional perspective on substance use among young people, covering both adolescents and youths, in SSA. This review provides the most recent evidence on the state of substance use among young people in SSA.

The pooled lifetime, 12-month, and current prevalence of any substance use among young people in SSA were 21%, 18%, and 15%, respectively. In general, the pooled prevalence estimated by the current review across different timeframes is lower than the overall prevalence of substance use reported in previous African and non-African studies. For instance, a recent review on substance use among adolescents in SSA reported a lifetime prevalence of 42% (10). Similarly, a review among medical students in India found an overall lifetime prevalence of 40% (98), and a study among young people in Ethiopia reported a 32% lifetime prevalence and a 24% current prevalence (99). Several factors could explain this difference, including the number of studies (60 in our review compared to fewer than 50 in others), the total number of participants (84,434 in our review compared to fewer in others), the characteristics of study participants (such as medical students in India who may use substances to cope with academic stress). Additionally, the scope of substances considered (we included various substances, while some studies focused on fewer substances, like those in Ethiopia focusing only on alcohol, khat, and cigarettes), and the limited geographical focus (Ethiopia and India specifically) contribute to the differences. Other factors, such as study periods, levels of stigma, potential response bias, and other methodological, cultural, and socioeconomic factors, may also justify the observed differences.

In this study, the 12-month and current alcohol prevalence rates, at 30% and 24% respectively, closely align with findings from previous studies. Specifically, our findings are consistent with the 12-month and current alcohol prevalence rates among young people in East Africa (26% and 28%) (100), alcohol prevalence among adolescents in SSA (32%) (10), and current alcohol prevalence among students in Ethiopia (27.6%) (101). Additionally, our results align with the alcohol prevalence among medical students in India (27%) (98), the lifetime (10%) and 12-month (5%) prevalence of cannabis among adolescents in SSA (102), and the current prevalence of khat among young individuals in Ethiopia (17.3%) (103). However, the lifetime prevalence reported in this review (21%) contrasts significantly with findings from other reviews on substance use among young people: Ethiopia (52%) (104), East Africa (52%) (100), students in India (40%) (98), and adolescents in Sub-Saharan Africa (SSA) (42%) (10).

Regionally, the lifetime and 12-month prevalence of any substance use were higher in the southern region, while the current prevalence was higher in the East Africa region. In country-specific analysis, Zambia had the highest lifetime prevalence of any substance use at 40%, Botswana had the highest 12-month prevalence at 32%, and Uganda had the highest current prevalence at 75%. This varying pattern of substance use prevalence among young people across study regions and countries within SSA could be attributed to the availability and variety of substances across regions as well as the number of studies involved from each region. Additionally, the differences could be explained by factors such as production, availability, cost, regulations, social pressure, and urban/rural distinctions (105, 106). Furthermore, distinct patterns of substance use observed between countries, such as the exclusive focus on khat in East Africa, highlight its origin and widespread consumption in countries like Ethiopia, Somalia, and Kenya (106–108). Similarly, the higher prevalence of cannabis reported in South Africa (32), aligns with the existence of indigenous plants of cannabis (known as dagga in Southern Africa) that have been used in traditional cultures for centuries (106). The relatively lower prevalence observed in the Western African region could be explained by the strong regional drug control and prevention policies and regulations (109), upon which Eastern and Southern Africa based their establishment of the Commission for Drug Control (ESACD).

This systematic review findings align with Social Norms Theory (110), which stipulates that people’s behaviors are influenced by the norms and expectations of their social groups. This theory helps explain why substance use prevalence varies across regions and countries in SSA, where cultural acceptance of substance use may be more pronounced. In environments where substance use is perceived as normative among peers, young individuals may feel pressure to engage in such behaviors to fit in (111).

The review identified that family dynamics, including positive reinforcement or discouragement of substance use, significantly shape young people’s attitudes and behaviors toward substance use. Additionally, family communication, parental monitoring, and parent-child relationships were found to have associations with substance use patterns. Social Learning Theory (Bandura, 1977) also complements these findings by suggesting that individuals, particularly young people, learn behaviors through observation and imitation of influential figures in their lives. Exposure to substance use behaviors among parents or older siblings normalizes these behaviors and increases the likelihood of experimentation and continued use during adolescence and young adulthood. Beyond family influences, it was reported that peer relationships, community norms, and broader societal factors further shape young people’s perceptions and behaviors related to substance use. Gender-disaggregated data of all specific substances from meta-analysis consistently show higher prevalence rates of substance use among males compared to females. For instance, lifetime alcohol use among males was 45.9% compared to 25.6% among females. Studies in Ethiopia and Europe similarly report higher overall substance use prevalence among male adolescents (99, 112, 113). The variations could arise from biological and socio-cultural factors such as childcare responsibilities, addiction stigma, relationship dynamics, peer pressure, group affiliations, and cultural norms (12, 99, 114).

Beyond the context of substance use variation by gender, the theories explain that children may observe their parents or older siblings using substances, which can normalize such behaviors and increase the likelihood of experimentation and continued use in adolescence and young adulthood. This observational learning process is particularly influential during developmental stages when individuals are forming their attitudes and behaviors toward substances (115, 116).

Moreover, family dynamics play a crucial role in shaping attitudes and behaviors related to substance use (117). Positive reinforcement of substance use behaviors within the family, whether overt or subtle, can reinforce these behaviors as acceptable or even desirable. Conversely, negative attitudes or behaviors towards substance use within the family unit can serve as protective factors against substance use initiation or escalation. Family communication patterns, parental monitoring, and the quality of parent-child relationships also were identified as significantly associated with the likelihood of substance use among young people.

In addition to family influences, peer relationships, community norms, and broader societal factors further shape young people’s perceptions and behaviors related to substance use (118).

Another important finding identified by this review is the gap in community-based substance use studies compared to those conducted in institutional settings. Out of the 60 studies reviewed, only about one-quarter involved participants were from the general community, while the rest focused on institutional settings. Community-based studies revealed significantly higher current prevalence rates (26%) than institution-based studies while showing similar lifetime rates. These findings underscore the critical importance of expanding research efforts to include young people from the general community in studies on substance use, to comprehensively understand the prevalence and factors influencing substance use in different contexts, and to develop targeted interventions that address the needs of all young people.

The current review also revealed a relative increase in publication trends on substance use over the last decade with a notable rise in the past five years in SSA. This increase, particularly during the COVID-19 era, might be attributed to an increased focus on research and write-up during the lockdown period. The lockdowns provided researchers with more time to conduct literature reviews, analyze data, and write manuscripts, potentially leading to a significant rise in academic output in this field. The pandemic may also have exacerbated substance use due to heightened stress, anxiety, and disruption of social and economic activities, thereby prompting more research interest in this area. This was supported by studies showing that there was a sharp increase in the publication of articles on different subjects (119, 120).

## Strength and limitations

This systematic review synthesized and analyzed a large body of empirical evidence from various studies, providing a comprehensive overview of substance use prevalence and associated factors across the region by using a systematic approach. By including studies covering a wide age range (10-24 years) and various types of substances, the review offers a more thorough understanding of substance use prevalence among young individuals in SSA across different reporting timeframes. It also highlights the differences in substance use prevalence across countries, regions, and genders, emphasizing the need for targeted interventions sensitive to cultural norms and practices.

However, this review has some limitations. Only cross-sectional studies published in peer-reviewed literature were included, missing grey literature such as government reports and unpublished data. This focus on epidemiological studies may have missed valuable insights from other sources. Although we strictly followed PRISMA guidelines, the inclusion of cross-sectional studies could introduce recall bias. Additionally, we observed higher heterogeneity in the review. While the JBI-MAStARI tool is valuable for assessing study quality, we acknowledge that its subjective nature and reliance on evaluator interpretation may introduce bias.

## Implications of study findings

The findings of this systematic review and meta-analysis have several important implications. The high prevalence rates of alcohol, cigarette, khat, and cannabis use among young people underscore the need for targeted interventions and policies. Identifying individual, family, and community risk factors provides a comprehensive understanding of the determinants of substance use, which is essential for designing multifaceted prevention strategies. Significant variations in substance use prevalence by gender, region, country, and study setting call for specific prevention and treatment approaches. Early screening and identification of substance use in healthcare and educational settings are crucial for timely intervention.

## Conclusions and recommendations

A significant portion of young people in SSA use different substances, at different time points with variations between genders, regions, and countries. Review findings highlight the need for interventions targeting both the broader young population and specific subgroups who may be at higher risk of substance use. Promotive, protective, and curative programs for substance use and substance use disorders in young people at the individual, family, and societal levels can play a key role in achieving sustainable development goals for their health and well-being.

Future longitudinal studies are crucial to understanding the progression of substance use and identifying causal relationships. Exploring regional and cultural differences in substance use patterns and including diverse populations beyond educational institutions, such as those in rural areas and marginalized groups, is also critical. Policy development should focus on targeted prevention programs for high-risk groups, such as males, older adolescents, and those with poor academic performance. Increasing school-based prevention and intervention programs is critical for early detection. Integrating substance use prevention with mental health services and engaging communities in prevention efforts can effectively address local risk factors. Routine screening in healthcare and educational settings, comprehensive substance use education, family engagement, strengthening parent-child relationships, peer support programs, and improved access to youth-friendly and culturally appropriate treatment and counseling services are vital practical measures.

## Data Availability

The original contributions presented in the study are included in the article/**Supplementary Material**. Further inquiries can be directed to the corresponding author/s.
