# Supplementary material for: Substance use among young people in sub-Saharan Africa: a systematic review and meta-analysis
Source: Front Psychiatry. 2024 Sep 11;15:1328318. doi: 10.3389/fpsyt.2024.1328318 (PMC11422104; doi:10.3389/fpsyt.2024.1328318)
Supplement: Supplementary file 1 [file DataSheet1.zip › S1_Search terms.docx]

# **Search term and strategy**

# **Search term**

## ***Concept 1: Substance use***

[“substance *use”/, “substance dependence”/, “substance consumption”/, “substance use disorder”/, “drug *use”/, “drug dependence”, addict*/, “alcohol *use”/, “alcohol drinking”, alcoholism/, “alcohol use disorder”/, “hazardous drinking”/, “harmful drinking”/, “khat *use”/, “chat *use”/, “khat dependence”/, “khat consumption”/, “cigar* smok*”/, nicotine/, “psychoactive substance use”/, “cannabis *use”/, “marijuana *use”/, “cocaine use”, “opioid use”]

## ***Concept 2: Prevalence***

[prevalence/, magnitude/, epidemiology/, proportion/]

## ***Concept 3: Young people***

[“young people”/, young*/, youth/, adolescen*/, underage/, teenage*/, student/]

## ***Concept 4: Geographical coverage****:*

[sub-Saharan Africa- list of countries in the sub-Saharan region, separated by the Boolean term “OR” was included in the search]

**Search Strategy:** Combine all concepts using Boolean operators

- Concept 1 AND Concept 2 AND Concept 3 AND Concept 4

Table: PICO elements for research on substance use among young people in sub-Saharan Africa

| **PICO elements** | **Detail description** |
| --- | --- |
| Population | Young people aged from 10-24 years (adolescent and youth), living in sub-Saharan Africa |
| Intervention | Various forms of substance use, including alcohol, khat, cigarette, cannabis, cocaine, shisha, opioids, and others |
| Comparison | Basically, comparisons may involve different characteristics (e.g., gender, regional differences within sub-Saharan Africa, study setting and year of publication). |
| Outcome | Prevalence and associated factors of substance use among young people |
