# Supplementary material for: Substance use among young people in sub-Saharan Africa: a systematic review and meta-analysis
Source: Front Psychiatry. 2024 Sep 11;15:1328318. doi: 10.3389/fpsyt.2024.1328318 (PMC11422104; doi:10.3389/fpsyt.2024.1328318)
Supplement: Supplementary file 1 [file DataSheet1.zip › S2_The JBI Criteria Used in methodological Quality Assessment.docx]

**The JBI Criteria Used in methodological Quality Assessment**

| **Items** | **Yes** | **No** |
| --- | --- | --- |
| 1. Was the sample frame appropriate to address the target population? |  |  |
| 1. Were study participants sampled in an appropriate way? |  |  |
| 1. Was the sample size adequate? |  |  |
| 1. Were the study subjects and the setting described in detail? |  |  |
| 1. Was the data analysis conducted with sufficient coverage of the identified sample? |  |  |
| 1. Were valid methods used for the identification of the condition? |  |  |
| 1. Was the condition measured in a standard, reliable way for all participants? |  |  |
| 1. Was there appropriate statistical analysis? |  |  |
| 1. Was the response rate adequate, and if not, was the low response rate managed appropriately? |  |  |

Yes= 1 No= 0
