# Supplementary material for: Substance use among young people in sub-Saharan Africa: a systematic review and meta-analysis
Source: Front Psychiatry. 2024 Sep 11;15:1328318. doi: 10.3389/fpsyt.2024.1328318 (PMC11422104; doi:10.3389/fpsyt.2024.1328318)
Supplement: Supplementary file 1 [file DataSheet1.zip › S3_Quality Appraisal Table.docx]

**Supplementary Table 2: Quality appraisal status of studies included in the systematic review and meta-analysis according to JBI characteristics**

| **S.#** | **Author, Year** | **Study design** | **Q1** | **Q2** | **Q3** | **Q4** | **Q5** | **Q6** | **Q7** | **Q8** | **Q9** | **Total per each Item** |
| --- | --- | --- | --- | --- | --- | --- | --- | --- | --- | --- | --- | --- |
| 1 | Ogunkunle et al., 2020 | Cross-sectional | 1 | 1 | 1 | 1 | 0 | 0 | 1 | 1 | 1 | 7 |
| 2 | Gobopamang et al., 2016 | Cross-sectional | 0 | 1 | 0 | 1 | 1 | 0 | 1 | 1 | 1 | 6 |
| 3 | Teni et al., 2015 | Cross-sectional | 1 | 1 | 1 | 0 | 0 | 0 | 1 | 1 | 1 | 6 |
| 4 | Ofonime et al., 2017 | Cross-sectional | 1 | 0 | 1 | 1 | 0 | 0 | 1 | 1 | 1 | 6 |
| 5 | Hamdulay AK., & Mash R., 2011 | Cross-sectional | 1 | 0 | 0 | 1 | 0 | 0 | 1 | 1 | 1 | 5 |
| 6 | Kassa et al., 2014 | Cross-sectional | 1 | 1 | 1 | 1 | 1 | 1 | 1 | 1 | 1 | 9 |
| 7 | Siziya et al., 2013 | Cross-sectional | 1 | 1 | 1 | 1 | 1 | 1 | 0 | 1 | 0 | 7 |
| 8 | Dida et al., 2014 | Cross-sectional | 1 | 1 | 1 | 0 | 1 | 0 | 0 | 1 | 1 | 6 |
| 9 | Abdeta et al., 2017 | Cross-sectional | 1 | 1 | 1 | 1 | 1 | 0 | 1 | 1 | 1 | 8 |
| 10 | Haleem A. et al., 2019 | Cross-sectional | 1 | 1 | 0 | 0 | 1 | 1 | 0 | 0 | 1 | 5 |
| 11 | Tsegaye A. et al., 2021 | Cross-sectional | 1 | 1 | 1 | 1 | 1 | 0 | 0 | 1 | 1 | 7 |
| 12 | Bright A. et al., 2016 | Cross-sectional | 1 | 1 | 0 | 1 | 0 | 0 | 1 | 1 | 0 | 5 |
| 13 | Adebiyi et al., 2010 | Cross-sectional | 1 | 1 | 0 | 1 | 0 | 0 | 0 | 0 | 1 | 4 |
| 14 | Adere et al., 2017 | Cross-sectional | 1 | 1 | 1 | 1 | 1 | 0 | 0 | 1 | 1 | 7 |
| 15 | Admasu et al., 2018 | Cross-sectional | 1 | 1 | 1 | 1 | 1 | 0 | 0 | 0 | 1 | 6 |
| 16 | Ajayi et al., 2019 | Cross-sectional | 1 | 0 | 0 | 1 | 1 | 0 | 0 | 1 | 0 | 4 |
| 17 | Ajayi et al., 2020 | Cross-sectional | 0 | 1 | 0 | 1 | 0 | 0 | 1 | 0 | 1 | 4 |
| 18 | Alebachew et al., 2019 | Cross-sectional | 1 | 1 | 1 | 1 | 1 | 1 | 1 | 1 | 1 | 9 |
| 19 | Astatkie et al., 2015 | Cross-sectional | 1 | 1 | 1 | 1 | 1 | 1 | 1 | 1 | 1 | 9 |
| 20 | Atwoli et al., 2011 | Cross-sectional | 0 | 0 | 0 | 1 | 0 | 1 | 1 | 0 | 0 | 3 |
| 21 | Ayenew et al., 2020 | Cross-sectional | 0 | 1 | 1 | 0 | 1 | 0 | 0 | 1 | 1 | 5 |
| 22 | Birhanu et al., 2014 | Cross-sectional | 1 | 1 | 1 | 1 | 1 | 0 | 0 | 1 | 1 | 7 |
| 23 | Weybright et al., 2018 | Cross-sectional | 1 | 1 | 0 | 1 | 0 | 0 | 0 | 1 | 0 | 4 |
| 24 | Chekole, 2020 | Cross-sectional | 1 | 1 | 1 | 1 | 1 | 0 | 1 | 1 | 1 | 8 |
| 25 | Chivandire, 2016 | Cross-sectional | 1 | 0 | 0 | 1 | 1 | 0 | 0 | 1 | 1 | 5 |
| 26 | Cumber, 2016 | Cross-sectional | 0 | 0 | 0 | 1 | 0 | 0 | 0 | 1 | 1 | 3 |
| 27 | Deressa, 2011 | Cross-sectional | 1 | 1 | 1 | 1 | 1 | 0 | 1 | 1 | 1 | 8 |
| 28 | Desai, 2019 | Cross-sectional | 1 | 1 | 1 | 1 | 1 | 0 | 1 | 1 | 1 | 8 |
| 29 | Dires et al., 2016 | Cross-sectional | 1 | 1 | 1 | 0 | 0 | 0 | 1 | 1 | 1 | 6 |
| 30 | Durowade et al., 2019 | Cross-sectional | 1 | 1 | 1 | 1 | 1 | 0 | 0 | 1 | 1 | 7 |
| 31 | Francis et al., 2015 | Cross-sectional | 1 | 1 | 1 | 1 | 1 | 1 | 1 | 1 | 1 | 9 |
| 32 | Gebrehanna et al., 2014 | Cross-sectional | 1 | 1 | 1 | 1 | 0 | 0 | 0 | 1 | 1 | 6 |
| 33 | Gebremariam et al., 2018 | Cross-sectional | 1 | 0 | 1 | 0 | 1 | 0 | 1 | 1 | 1 | 6 |
| 34 | Gebresilassie et al., 2020 | Cross-sectional | 1 | 1 | 1 | 0 | 1 | 1 | 1 | 1 | 1 | 8 |
| 35 | Gebreslassie et al., 2013 | Cross-sectional | 1 | 1 | 1 | 0 | 1 | 0 | 0 | 1 | 1 | 6 |
| 36 | Getachew et al., 2019 | Cross-sectional | 1 | 0 | 1 | 0 | 1 | 0 | 0 | 1 | 1 | 5 |
| 37 | Hirpa et al., 2021 | Cross-sectional | 1 | 0 | 1 | 1 | 1 | 0 | 1 | 1 | 1 | 7 |
| 38 | Ipingbemi et al., 2021 | Cross-sectional | 0 | 1 | 1 | 0 | 0 | 0 | 1 | 0 | 1 | 4 |
| 39 | Itanyi et al., 2020 | Cross-sectional | 1 | 1 | 1 | 0 | 1 | 1 | 1 | 0 | 1 | 7 |
| 40 | Kanyoni et al. 2015 | Cross-sectional | 1 | 1 | 1 | 1 | 1 | 1 | 1 | 1 | 1 | 9 |
| 41 | Kassa et al., 2017 | Cross-sectional | 1 | 1 | 1 | 1 | 1 | 0 | 1 | 1 | 1 | 8 |
| 42 | Kassa et al., 2016 | Cross-sectional | 1 | 1 | 1 | 1 | 1 | 0 | 1 | 1 | 1 | 8 |
| 43 | Kuteesa et al., 2020 | Cross-sectional | 1 | 1 | 1 | 1 | 1 | 1 | 1 | 1 | 1 | 9 |
| 44 | Lakew et al., 2014 | Cross-sectional | 1 | 0 | 1 | 0 | 1 | 0 | 0 | 0 | 1 | 4 |
| 45 | Manyike et al., 2016 | Cross-sectional | 0 | 0 | 0 | 0 | 1 | 0 | 1 | 1 | 0 | 3 |
| 46 | Mayanja et al., 2020 | Cross-sectional | 0 | 0 | 0 | 1 | 1 | 1 | 1 | 1 | 0 | 5 |
| 47 | Mokwena et al., 2021 | Cross-sectional | 1 | 0 | 1 | 0 | 0 | 0 | 1 | 0 | 1 | 4 |
| 48 | Moodley et al., 2012 | Cross-sectional | 0 | 0 | 0 | 1 | 0 | 0 | 1 | 0 | 1 | 3 |
| 49 | Mossie et al., 2015 | Cross-sectional | 0 | 0 | 1 | 1 | 1 | 0 | 0 | 0 | 1 | 4 |
| 50 | Musyoka et al., 2020 | Cross-sectional | 1 | 1 | 1 | 0 | 1 | 1 | 1 | 1 | 1 | 8 |
| 51 | Mutiso et al., 2022 | Cross-sectional | 1 | 1 | 1 | 1 | 1 | 1 | 1 | 1 | 1 | 9 |
| 52 | Ogunsola et al., 2016 | Cross-sectional | 1 | 1 | 1 | 1 | 1 | 0 | 1 | 1 | 1 | 8 |
| 53 | Olashore et al., 2018 | Cross-sectional | 1 | 0 | 0 | 1 | 1 | 0 | 1 | 1 | 1 | 6 |
| 54 | Onifade et al., 2014 | Cross-sectional | 0 | 0 | 0 | 1 | 1 | 0 | 1 | 1 | 0 | 4 |
| 55 | Onya et al., 2012 | Cross-sectional | 0 | 1 | 0 | 1 | 1 | 0 | 1 | 1 | 1 | 6 |
| 56 | Onyekachi-Chigbu et al., 2021 | Cross-sectional | 0 | 0 | 1 | 0 | 0 | 0 | 1 | 1 | 1 | 4 |
| 57 | Oshodi et al., 2010 | Cross-sectional | 1 | 1 | 1 | 0 | 1 | 0 | 1 | 1 | 1 | 7 |
| 58 | Owusu-Sarpong et al., 2019 | Cross-sectional | 0 | 1 | 0 | 1 | 1 | 0 | 0 | 1 | 1 | 5 |
| 59 | PLZwane et al., 2022 | Cross-sectional | 0 | 0 | 1 | 0 | 0 | 0 | 1 | 1 | 1 | 4 |
| 60 | Reda et al., 2012 | Cross-sectional | 1 | 1 | 1 | 1 | 0 | 0 | 1 | 1 | 1 | 7 |
| 61 | Riva et al., 2018 | Cross-sectional | 1 | 1 | 1 | 1 | 1 | 1 | 1 | 1 | 1 | 9 |
| 62 | Roble et al., 2021 | Cross-sectional | 1 | 1 | 1 | 0 | 0 | 0 | 1 | 1 | 1 | 6 |
| 63 | Seid et al., 2021 | Cross-sectional | 1 | 1 | 1 | 0 | 0 | 0 | 1 | 1 | 1 | 6 |
| 64 | Shegute et al., 2021 | Cross-sectional | 1 | 1 | 1 | 0 | 0 | 0 | 1 | 1 | 1 | 6 |
| 65 | Sinshaw et al., 2014 | Cross-sectional | 1 | 1 | 1 | 0 | 1 | 0 | 0 | 1 | 1 | 6 |
| 66 | Soepnel et al., 2022 | Cross-sectional | 1 | 0 | 0 | 1 | 1 | 1 | 1 | 1 | 0 | 6 |
| 67 | Soremekun et al., 2020 | Cross-sectional | 1 | 0 | 1 | 1 | 0 | 0 | 1 | 1 | 1 | 6 |
| 68 | Soremekun et al., 2021 | Cross-sectional | 0 | 1 | 1 | 0 | 0 | 0 | 1 | 0 | 1 | 4 |
| 69 | Tshitanganoet al., 2016 | Cross-sectional | 0 | 0 | 1 | 0 | 0 | 0 | 0 | 1 | 1 | 3 |
| 70 | Vorster et al., 2019 | Cross-sectional | 0 | 0 | 1 | 0 | 0 | 0 | 0 | 1 | 1 | 3 |
| 71 | Zeleke et al., 2013 | Cross-sectional | 0 | 1 | 1 | 0 | 0 | 0 | 0 | 1 | 1 | 4 |
| 72 | Augustus et ai., 2024 | Cross-sectional | 1 | 0 | 0 | 1 | 1 | 1 | 1 | 1 | 1 | 7 |
| 73 | Einarsdóttir, 2024 | Cross-sectional | 1 | 1 | 1 | 1 | 1 | 1 | 1 | 1 | 1 | 9 |
| 74 | Jaguga et al., 2023 | Cross-sectional | 1 | 0 | 0 | 1 | 1 | 1 | 1 | 1 | 1 | 7 |
| 75 | Kinyanjui et al., 2023 | Cross-sectional | 1 | 1 | 1 | 1 | 1 | 1 | 1 | 1 | 1 | 9 |
| 76 | Kyei-Gyamfi, 2024 | Cross-sectional | 1 | 1 | 1 | 1 | 1 | 1 | 1 | 1 | 1 | 9 |
| 77 | Kyei-Gyamfi, 2023 | Cross-sectional | 1 | 1 | 1 | 1 | 1 | 1 | 1 | 1 | 1 | 9 |
| 78 | Mavura, 2022 | Cross-sectional | 1 | 1 | 1 | 1 | 1 | 1 | 1 | 1 | 1 | 9 |
| 79 | Olashore, 2022 | Cross-sectional | 1 | 1 | 1 | 1 | 1 | 1 | 1 | 1 | 1 | 9 |
